# The Notification of Infectious Diseases

**Published:** 1899-09-16

**Authors:** 


### THE NOTIFICATION OF INFECTIOUS DISEASES.

A SMALL but important amendment of the Public Health Acts has been passed by Parliament in the late session. By the Infectious Diseases (Notification) Extension Act, 1899, the provisions of the Infectious Diseases Notification Act, 1889, have been extended to all urban, rural, and port sanitary districts in England and Wales. The Act of 1889 was merely adoptive; that is to say, it only operated in those districts in which it had been adopted by the sanitary authority. In these districts the Act required notice of any of the infectious diseases mentioned therein to be given to the local authority by the medical practitioner attending the infected patient, and also by the head of the family to which such patient belonged; or, in his default, by the patient's nearest relative; or, in their default, by every person in charge of or in attendance upon the patient; or in default of any such person, the master of the house. These provisions will now be compulsory in all districts. The metropolis is controlled in this respect by the similar but more precise enactments contained in the London Public Health Act, 1891. The new Act does not make any attempt to extend the list of infectious diseases, which still remains as defined by the previous Acts, viz., it includes small-pox, cholera, diphtheria, membraneous croup, erysipelas, scarlet fever or scarlatina, and typhus, typhoid, enteric, relapsing, continued, or puerperal fever; but the local authority in any district will be able by resolution and order, approved by the Local Government Board, to add to the list other diseases which they may be of opinion should be brought within the operation of the Act. In view of a recent case arising out of a death from consumption, it will be interesting to learn when local authorities will consider this disease should be scheduled. The new Act comes into force on January 1st, 1900. It may be added that in cases of lead, phosphorus, or arsenical poisoning or anthrax in factories and workshops, their notification to the Home Office is provided for by the Factory and Workshops Act, 1895.

